# Appointment in Michigan University and Admission of Women

**Published:** 1871-07

**Authors:** 


### Appointment in Michigan University and Admission of Women.

We learn that Prof. Theodore A. McGraw, of the Detroit Medical College has recently been elected Lecturer on Surgery in the Michigan University. We congratulate Michigan University on its being able to obtain the services of a man so eminently fitted for the position.

By virtue of the resolution of the Board of Regents opening the University to women, one young lady was admitted to the Academic Department at the close of the last year. At the beginning of the present year women were received for the first time into all the departments of the institution. The whole number of female students registered is thirty-four, two in the Law Department, eighteen in the Medical, and fourteen in the Department of Science, Literature and the Arts, the latter being distributed as follows: Three in the classical course, five in the Latin-scientific. one in the scientific, two in selected studies and three in the course of pharmacy. One has already graduated in law, one in medicine and two in pharmacy.

